# Antegrade thoracic endovascular aortic repair using an ascending aortofemoral through-and-through wire technique for a severely tortuous aorta associated with rickets

**DOI:** 10.1186/s40792-017-0324-0

**Published:** 2017-03-24

**Authors:** Atsushi Morishita, Kazuhiko Hanzawa, Seiichiro Katahira, Takeshi Hoshino, Hideyuki Tomioka

**Affiliations:** 1 The Department of Cardiovascular Surgery, Numata Neurosurgery Heart-Disease Hospital, 8 Sakae-cho, Numata, 378-0014 Japan; 2 The Department of Thoracic and Cardiovascular Surgery, Niigata University School of Medicine, Niigata, Japan; 3 The Department of Surgery, Tokyo Rosai Hospital, Oota-ku, Japan; 4 The Department of Anesthesiology, Minami Machida Hospital, Machida, Japan; 5 The Department of Cardiovascular Surgery, Heart Institute of Japan, Tokyo Women’s Medical University, Tokyo, Japan

**Keywords:** Thoracic endovascular aortic repair, An ascending aortofemoral through-and-through wire technique, Severe tortuous, Access route

## Abstract

**Background:**

Severe aortic tortuosity of the access route often prevents successful complete exclusion of an aneurysm in thoracic endovascular aortic repair (TEVAR).

**Case presentation:**

We performed antegrade TEVAR on a 79-year-old man with right hemiparesis. We deployed the stent graft from the ascending aorta with a tube graft conduit to treat a descending thoracic aortic aneurysm associated with rickets and multiple comorbidities. Although the application of a ministernotomy diminished the potential advantages of endovascular treatment in view of less invasive surgery, antegrade TEVAR using an ascending aortofemoral through-and-through wire technique was a good option in this patient because a conventional retrograde approach was not feasible due to his severely tortuous aorta.

**Conclusions:**

To avoid device-related complications, it is crucial to make a prudent preoperative decision on a patient-by-patient basis, taking into account the appropriate access site, adjuvant guidewire technique, and adjunctive surgical interventions.

## Background

Thoracic endovascular aortic repair (TEVAR) is increasingly popular for treating various thoracic aortic pathologies. Severe aortic tortuosity of the access route often necessitates alteration of the access site, the adoption of adjuvant guidewire techniques, and adjunctive surgical interventions for precise positioning and safe delivery of the stent graft. Antegrade TEVAR, which involves deployment of the stent graft from the ascending aorta with a tube graft conduit, is usually combined with aortic great vessel debranching for aortic arch pathologies. However, this remains a rare strategy for descending thoracic aortic pathologies [1].

In this report, we describe the use of antegrade TEVAR for a descending thoracic aortic aneurysm associated with rickets. In the present case, ascending aortofemoral through-and-through wire technique was effective for advancing the stent graft into a severely tortuous aorta.

## Case presentation

**Fig. 1:**
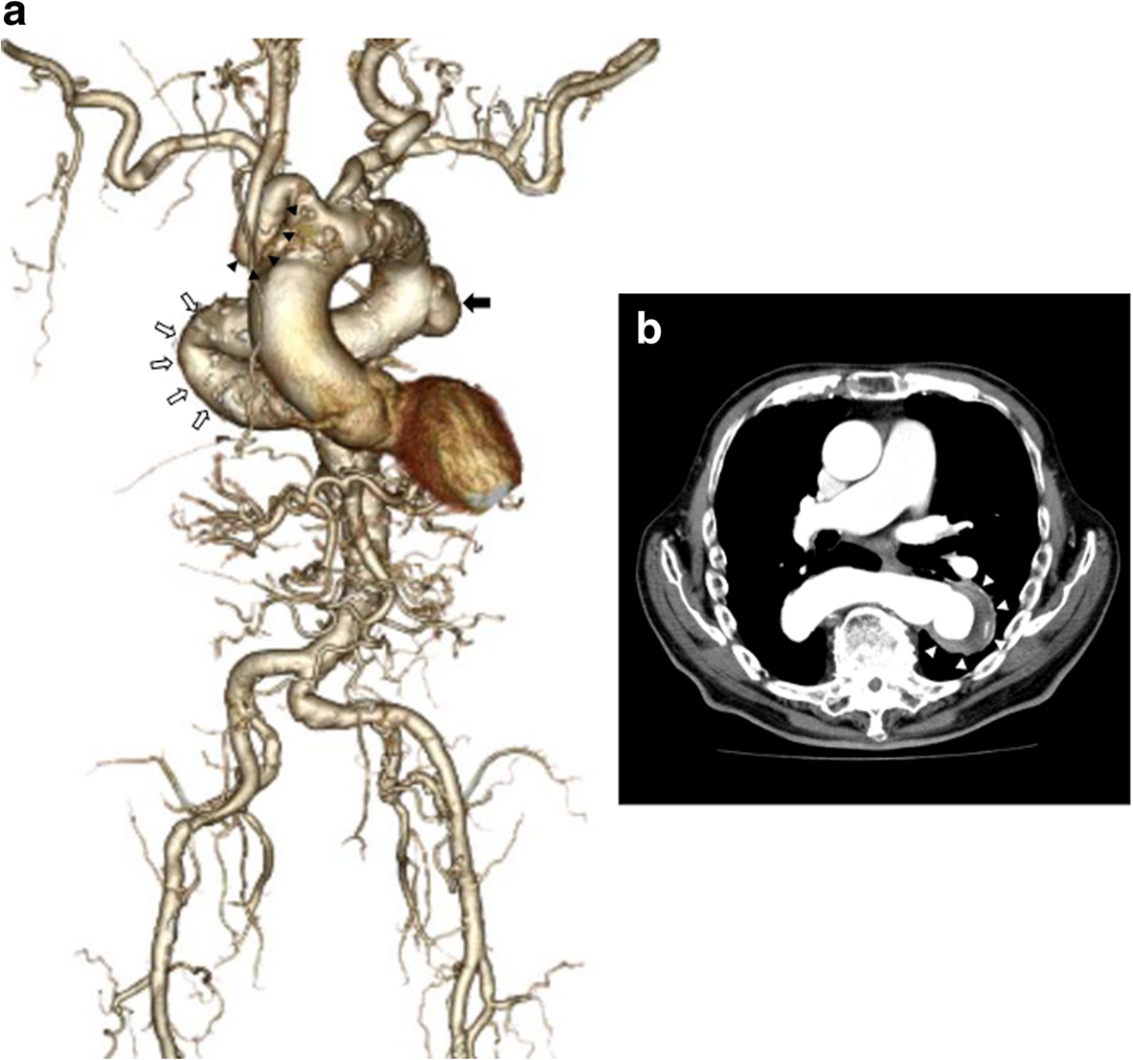
Preoperative computed tomography angiography. Preoperative computed tomography angiography demonstrating a saccular aneurysm of the descending thoracic aorta with a maximum diameter of 55 mm (**a** *black arrow*; **b** *white arrowheads*). The thoracoabdominal aorta adjoining the aneurysm possessed a bend of 180**°** (*white arrow*), and the right brachiocephalic artery was extremely elongated and tortuous (*black arrowheads*)

A 79-year-old man with right hemiparesis was admitted to our hospital for examination of a growing abnormal shadow on a chest radiograph. He had experienced rickets as a child and had been treated for hypertension, mild chronic obstructive pulmonary disease, cerebral infarction, and cerebral hemorrhage for 20 years. His height, weight, and body surface area were 133 cm, 44.2 kg, and 1.25 m^2^, respectively. He also had pigeon breast. Computed tomography (CT) angiography demonstrated a saccular aneurysm of the descending thoracic aorta with a maximum diameter of 55 mm. The thoracoabdominal aorta adjoining the aneurysm possessed a bend of 180°, and the right brachiocephalic artery was extremely elongated and tortuous (Fig. 1). Transthoracic echocardiography revealed moderate aortic valve regurgitation. Considering respiratory failure by left thoracotomy and his medical history such as cerebral infarction and cerebral hemorrhage, the patient was deemed unsuitable for performing a traditional graft replacement. After informed consent was obtained from the patient, we selected TEVAR under a ministernotomy.

Although the iliac arteries were suitable for the introduction of a sheath for the chosen stent graft, we were concerned that the tortuous thoracoabdominal aorta would prevent safe retrograde delivery of the stent graft to its intended location. The right brachiofemoral through-and-through wire technique was not feasible because of a high risk of vascular injury associated with the severely tortuous right brachiocephalic artery. The use of a transapical approach was considered difficult because of the hemodynamic collapse caused by aortic regurgitation. After considering the available options, we decided to deliver the stent graft from the ascending aorta, using the ascending aortofemoral through-and-through wire technique.

**Fig. 2:**
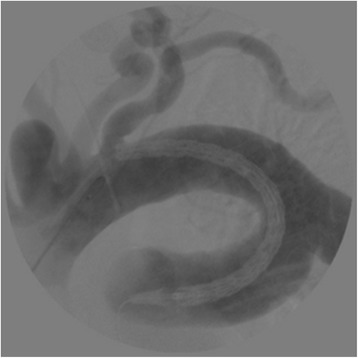
Intraoperative digital subtraction angiography. Intraoperative digital subtraction angiography demonstrating the stent graft positioned around the aneurysm

**Fig. 3:**
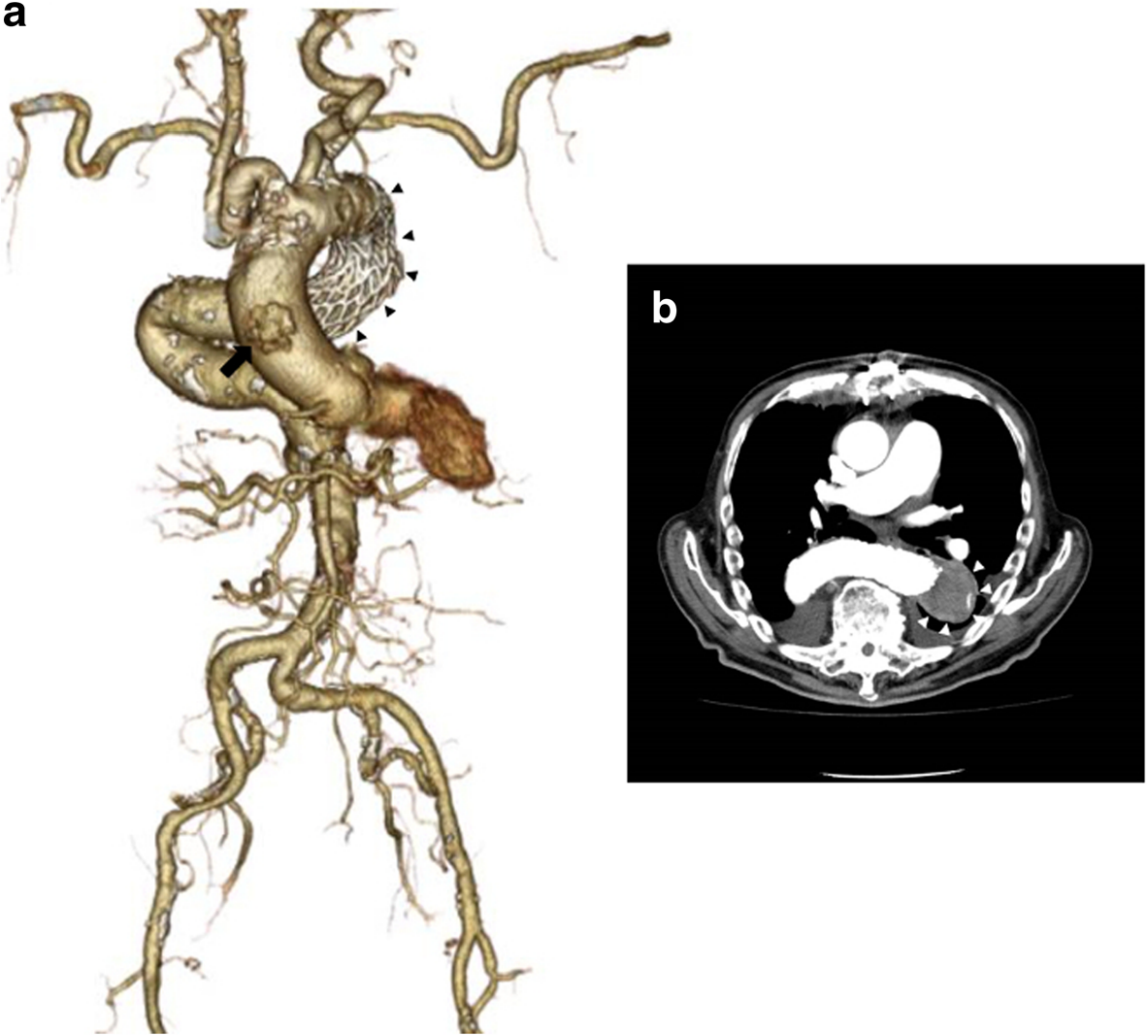
Postoperative computed tomographic angiography. Postoperative computed tomography angiography demonstrating the complete exclusion of a saccular thoracic aneurysm (**a** *black arrowheads*; **b** *white arrowheads*) and the stump of a prepared graft conduit (*black arrow*)

Under general anesthesia and transcranial Doppler and cerebral oximetry monitoring, we performed an inverted T ministernotomy below the second intercostal space. Because the diameter of the ascending aorta was >40 mm, we prepared the conduit in the ascending aorta under partial cardiopulmonary bypass. A 10-mm woven Dacron graft was anastomosed in an end-to-side fashion to the ascending aorta, using 4–0 monofilament sutures under partial clamping. A 5-Fr sheath was placed in the graft, and a 400-cm, 0.035-inch hydrophilic guidewire was inserted through a pigtail catheter to the descending aorta. The wire was snared within the abdominal aorta and externalized through the right common femoral artery. Subsequently, the 5-Fr sheath in the graft was exchanged to a 22-Fr sheath (dry seal sheath, W.L. Gore & Associates, Inc.). Aortography was performed using the pigtail catheter, which was introduced beside the stent graft into the large sheath. A 31 × 150-mm conformable GORE TAG stent graft (W.L. Gore & Associates, Inc., Flagstaff, AZ, USA), which was designed to be released from the middle position, was advanced in the antegrade direction across the aortic arch and subsequently deployed distal to the left subclavian artery by repeated pull-loosen technique with both sides of the wire (Fig. 2). No endoleak was detected by angiography after performing touch-up via inflation of a balloon (tri-lobe balloon catheter, W.L. Gore & Associates, Inc.). The patient’s postoperative course was uneventful and without major complications such as cerebrovascular accidents, vascular access issues, respiratory failure, cardiac failure, wound infection, or paraplegia. Postoperative CT angiography demonstrated complete exclusion of the saccular thoracic aneurysm (Fig. 3). The patient has been discharged from hospital and is now undergoing monthly follow-up examinations.

### Discussion

Currently, thoracic endovascular aortic repair is the first treatment of choice in patients with aneurysms of the descending thoracic aorta, depending on the condition of the patient, demographic factors, and suitable anatomy for stent-graft deployment. When there is an abnormal anatomical characteristic, such as severe aortic tortuosity, technical success may not be possible using ordinary maneuvers involving a stiff guidewire alone and delivery of stent grafts from femoral arteries in an antegrade direction. Furthermore, a catastrophic situation caused by thrombus or plaque embolization and rupture of calcifications can occur. In such anatomically challenging cases, it is important to carefully consider the choice of access route, the use of adjuvant guidewire technique, and the need for adjunctive surgical interventions. Several adjuvant techniques have been reported as means of overcoming these issues. The use of stiff “buddy” wires and the introduction of a coaxial sheath should be performed primarily to straighten an excessively tortuous aorta, but delivery of the stent graft can be difficult. The through-and-through wire technique, which pulls both sides of an externalized guidewire with adequate tension and straightens the severely tortuous aorta, is a powerful means of delivering stent grafts [2]. The through-and-through wire technique from the right brachial artery to the femoral artery was not feasible in the present case because the right brachiocephalic artery was severely tortuous [3]. The left common carotid artery and left subclavian artery did not allow the use of an externalized guidewire because of multiple severe calcifications around the supra-aortic vessels. Joseph et al. described a technique to create a transseptal externalized guidewire loop [4]. It was thought that this technique would result in acute deterioration of valve regurgitations caused by the indwelling guidewire. Furthermore, Saouti et al*.* described the efficacy of a transapical approach, which is often available in transcatheter aortic valve replacement [5]. This approach can lead to several complications such as respiratory failure by left thoracotomy, hemodynamic collapse due to aortic regurgitation, and ventricular pseudoaneurysm formation. Ultimately, we decided to perform antegrade TEVAR from the ascending aorta with a tube graft conduit.

Because the maximum diameter of the ascending aorta was >40 mm, we decided to prepare the conduit to prevent uncontrollable bleeding and unexpected aortic dissection. To reduce the risk of intraoperative stroke, we ensured that the tip of the large sheath did not damage the wall of the atherosclerotic aorta. Debranching of the supra-aortic vessels in combination with TEVAR is performed during median sternotomy and right anterior minithoracotomy. The right anterior minithoracotomy approach is appropriate in selected high-risk patients with prior cardiac surgery [6]. The application of a ministernotomy for preparing a conduit appeared to be a safe and reliable method, particularly in the present case with multiple comorbidities, although the potential advantage of endovascular treatment declined in the view of less invasive surgical options.

Chen et al*.* reported that high tortuosity of the thoracic aorta is associated with higher occurrence of endoleaks and lower survival in patients undergoing TEVAR for atherosclerotic aneurysms [7]. Moreover, it has been speculated that perioperative complications related to TEVAR show significant associations with tortuousity. It was conceivable that the use of a hydrophilic guidewire would contribute to the compatibility of stent graft placement for a tortuous aorta compared with the use of a stiff guidewire. Furthermore, it was considered that delicate manipulation by repeating pull-loosen with both sides of the hydrophilic guidewire could play an important role in minimizing the damage of the atherosclerotic aorta by the stent graft.

With respect to the precise deployment of the stent graft to its intended position, there are several adjuncts including right ventricular rapid pacing, administration of adenosine or vasodilators, balloon inflation in the inferior vena cava, and through-and-through bowing technique [8]. In addition, it was difficult to determine the accurate proximal sealing zone when performing the reversed deployment of the stent graft. Although the use of a hydrophilic guidewire prevented the stent graft from pressing against the greater curvature of the thoracic aorta, the longer stent graft coverage was thought to obviate migration of the stent graft and the occurrence of endoleaks by taking advantage of the anatomical characteristics in the present case, i.e., the aneurysm existed at the top of a sharply curved thoracic aorta.

## Conclusions

Antegrade TEVAR using an ascending aortofemoral through-and-through wire technique represents an alternative treatment option in patients in whom a conventional retrograde approach is not feasible due to the presence of a severely tortuous aorta. The present case highlights the importance of prudent, personalized preoperative decision making to prevent device-related complications.

## Abbreviations

CT: Computed tomography
TEVAR: Thoracic endovascular aortic repair
